# Identification of microRNAs and their response to the stress of plant allelochemicals in *Aphis gossypii* (Hemiptera: Aphididae)

**DOI:** 10.1186/s12867-017-0080-5

**Published:** 2017-02-16

**Authors:** Kang-Sheng Ma, Fen Li, Ying Liu, Ping-Zhuo Liang, Xue-Wei Chen, Xi-Wu Gao

**Affiliations:** China Agricultural University, Beijing, China

**Keywords:** MicroRNAs, Plant allelochemicals, *Aphis gossypii*, Illumina sequencing, Regulatory roles

## Abstract

**Background:**

MicroRNAs (miRNAs) are a group of short non-coding RNAs involved in the inhibition of protein translation or in mRNA degradation. Although the regulatory roles of miRNAs in various biological processes have been investigated, there is as yet an absence of studies about the regulatory roles of miRNAs involved in the metabolism of plant allelochemicals in insects.

**Results:**

We constructed five small RNA libraries from apterous *Aphis gossypii* adults that had fed on an artificial diet containing various allelochemicals. Using Illumina sequencing, a total of 73.27 million clean reads was obtained, and 292 miRNAs were identified from *A. gossypii*. Comparative analysis of read counts indicated that both conserved and novel miRNAs were differently expressed among the five libraries, and the differential expression was validated via qRT-PCR. We found that the transcript levels of several miRNAs were increased or decreased in all of the allelochemical treatment libraries compared to the control. The putative target genes of the miRNAs were predicted using in silico tools, and the target genes of several miRNAs were presumed to be involved in the metabolism of xenobiotic compounds. Furthermore, the target prediction results were confirmed using dual luciferase reporter assay, and Ago-miR-656a-3p was demonstrated to regulate the expression of *CYP6J1* post-transcriptionally through binding to the 3′ UTR of *CYP6J1*.

**Conclusion:**

Our research results indicate that miRNAs may be involved in the metabolism of plant allelochemicals in *A. gossypii*, and these results also represent an important new small RNA genomics resource for further studies on this topic.

**Electronic supplementary material:**

The online version of this article (doi:10.1186/s12867-017-0080-5) contains supplementary material, which is available to authorized users.

## Background

MicroRNAs (miRNAs) are small non-coding RNAs (18-24 nucleotides in length) that regulate gene expression at the post-transcriptional level [1, 2]. Through binding complementarily to 3′ untranslated regions (UTRs), coding sequences, or 5′ UTRs, miRNA suppress the translation of target mRNA molecules, and thereby silence target gene expression [3–5]. miRNAs are generated in all eukaryotes and viruses [1, 6], and many miRNAs are conserved among related species [7]. Since the first miRNA was reported to regulate the timing of development in *Caenorhabditis elegans* [8], numerous miRNAs have been identified from animals, plants, and viruses. In the last two decades, thousands of miRNAs have been isolated from insect species, including *Drosophila melanogaster* [9], *Bombyx mori* [10, 11], *Manduca sexta* [12–14], *Plutella xylostella* [15, 16], and *Helicoverpa armigera* [17, 18]. Functional studies carried out in these insect species have demonstrated that insect miRNAs play very important regulatory roles in various biological processes, such as development, immune responses, metabolism, and host-pathogen interactions [1, 6, 19, 20].

The cotton aphid, *Aphis gossypii* Glover (Hemiptera: Aphididae), is an important insect pest in cotton and cucurbit crops that causes economic damage both through direct feeding and through the transmission of viruses [21, 22]. Given that *A. gossypii* has a very wide host range that encompasses least 300 species [23, 24], this pest encounters multiple plant toxic chemicals that are produced by host plants to defend against herbivores. These compounds have strong deleterious effects on herbivorous insects by affecting the growth and development or even by directly causing mortality [25, 26]. In humans and large mammals, increasing evidence suggests that miRNAs play very important roles in the metabolism of xenobiotic compounds [5, 27]. Such miRNAs can mediate the detoxification metabolism of xenobiotics by regulating the expression of xenobiotic-metabolizing enzymes and nuclear receptors [5]. For example, human P450 CYP1A1, which is involved in the metabolism of carcinogenic metabolites, was found to be post-transcriptionally regulated by miR-892a [28]. In addition, previous studies carried out in mosquitoes *Culex pipiens* suggested that miRNAs participate in the resistance to pyrethroid insecticides by mediating the expression levels of P450 genes [29, 30]. While there are a lot known about the miRNAs that participate in regulating the detoxification of xenobiotics in animals and miRNAs likely have essential roles in insecticide resistance, less is understood about the regulatory roles of miRNAs in the metabolism of plant allelochemicals in insects.

In the present study, five small RNA libraries were built from apterous *A. gossypii* adults fed on artificial diets that contained various plant allelochemicals (gossypol, 2-tridecanone, quercetin and tannic acid respectively, which are toxic chemicals found naturally in cotton plant or other host plants of the cotton aphid) and control. A total of 73.27 million clean reads was obtained by deep sequencing, and 292 miRNAs were identified from the five sample libraries. In order to identify putative allelochemical metabolism-related miRNAs, the expression levels of both conserved and novel miRNAs were compared among the five libraries, and the targets of the newly identified miRNAs were predicted. The results of this study deepen our understanding of the regulatory roles of miRNAs in *A. gossypii* and indicate that miRNAs are likely involved in the insect metabolism of plant allelochemicals.

## Results

### Deep sequencing of *A. gossypii* small RNA libraries

**Fig. 1:**
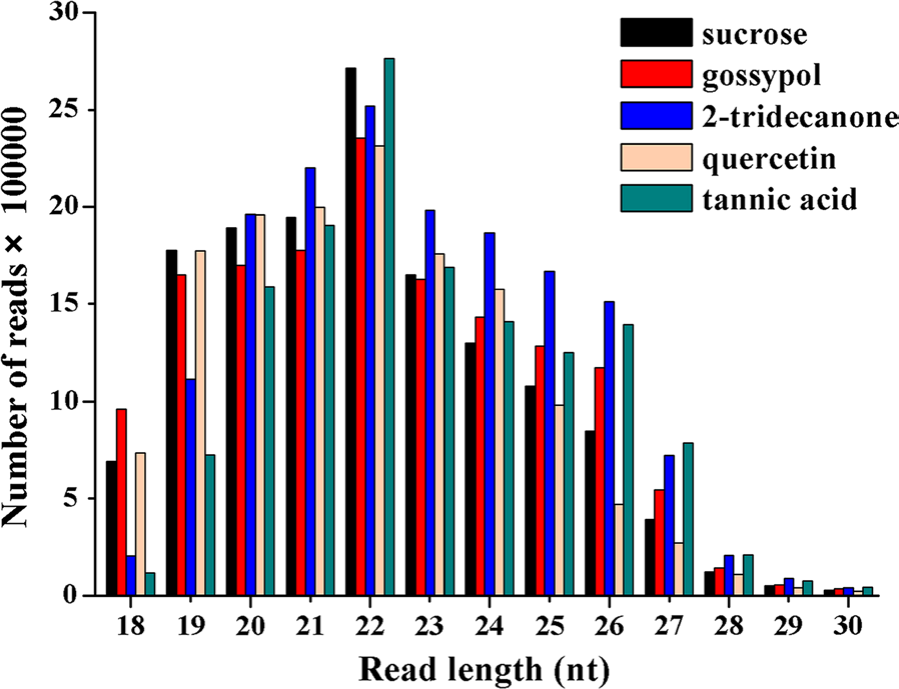
Length distribution of small RNAs from *A. gossypii* identified by deep sequencing. This length distribution was assessed using clean reads after filtering out the redundant small RNAs

In order to examine the potential role of small RNAs in *A. gossypii* responses to plant allechemicals, we collected apterous *A. gossypii* adults that had been fed artificial diet containing various allechemicals for 24 h for small RNA sequencing. Five *A. gossypii* small RNA libraries were constructed and sequenced using the Illumina sequencing platform. A total of 76.96 million raw reads was obtained from the five libraries, and after filtering out sequences shorter than 18 nt and filtering the lower quality reads from the raw data, 73.27 million quality reads were obtained. The number of clean reads differed among the five libraries; more clean reads (16.09 million) were obtained from the 2-tridecanone-fed library than from any of the other four libraries (14.48, 13.96, 14.01, and 14.74 million, in the tannic acid, quercetin, gossypol, and control libraries respectively) (Table 1). The length of these small RNAs ranged from 18 to 30 nt. In all five libraries, the highest peak for nucleotide length distribution was that for 22 nt (Fig. 1; Additional file 1).

**Table 1:** Total number of reads obtained from the small RNA libraries from aphids fed artificial diet containing various plant allechemicals

Allechemicals	Raw reads	Clean reads
Control	15,193,417	14,483,649
2-tridecanone	16,819,205	16,090,539
Tannic acid	14,630,011	13,956,679
Quercetin	14,743,194	14,006,538
Gossypol	15,575,107	14,736,872
Total	76,960,934	73,274,277

### Identification of conserved and novel miRNAs, and analysis of their features

To identify potential candidate miRNAs from the *A. gossypii* sample libraries, the raw sequencing data were analyzed with miRDeep2 software. The *Acyrthosiphon pisum* genome was used as a reference because the *A. gossypii* genome was not available at the time of the analysis. Total of 292 unique miRNA candidates were identified from among the five libraries, including 246 conserved miRNAs and 46 potentially novel miRNAs. The newly identified miRNAs of *A. gossypii* were named by prefixed with “Ago”, where “Ago” means *A. gossypii*. The length and copy number distribution analysis of these newly identified miRNAs indicated that the miRNAs of 22 nt in length were the most abundant (47.46%), and the miRNAs of 21–22 nt in length accounted for more than 81% of reads (Table 2). Further, we analyzed the common and unique distribution of the newly identified miRNAs from among the five libraries. The results demonstrated that 221 miRNAs were common among the five libraries; only a few of the miRNAs were uniquely expressed in a particular library (Additional file 2: Figure S1).

**Table 2:** Length distribution and copy number of *A. gossypii* miRNAs

miRNA length	Number of miRNAs	Copy number	Percentage (%)
18	65	11,291	0.32
19	64	62,839	1.79
20	40	100,111	2.85
21	37	1,183,236	33.69
22	54	1,666,826	47.46
23	17	309,952	8.82
24	11	178,019	5.07
25	4	31	0.00

**Fig. 2:**
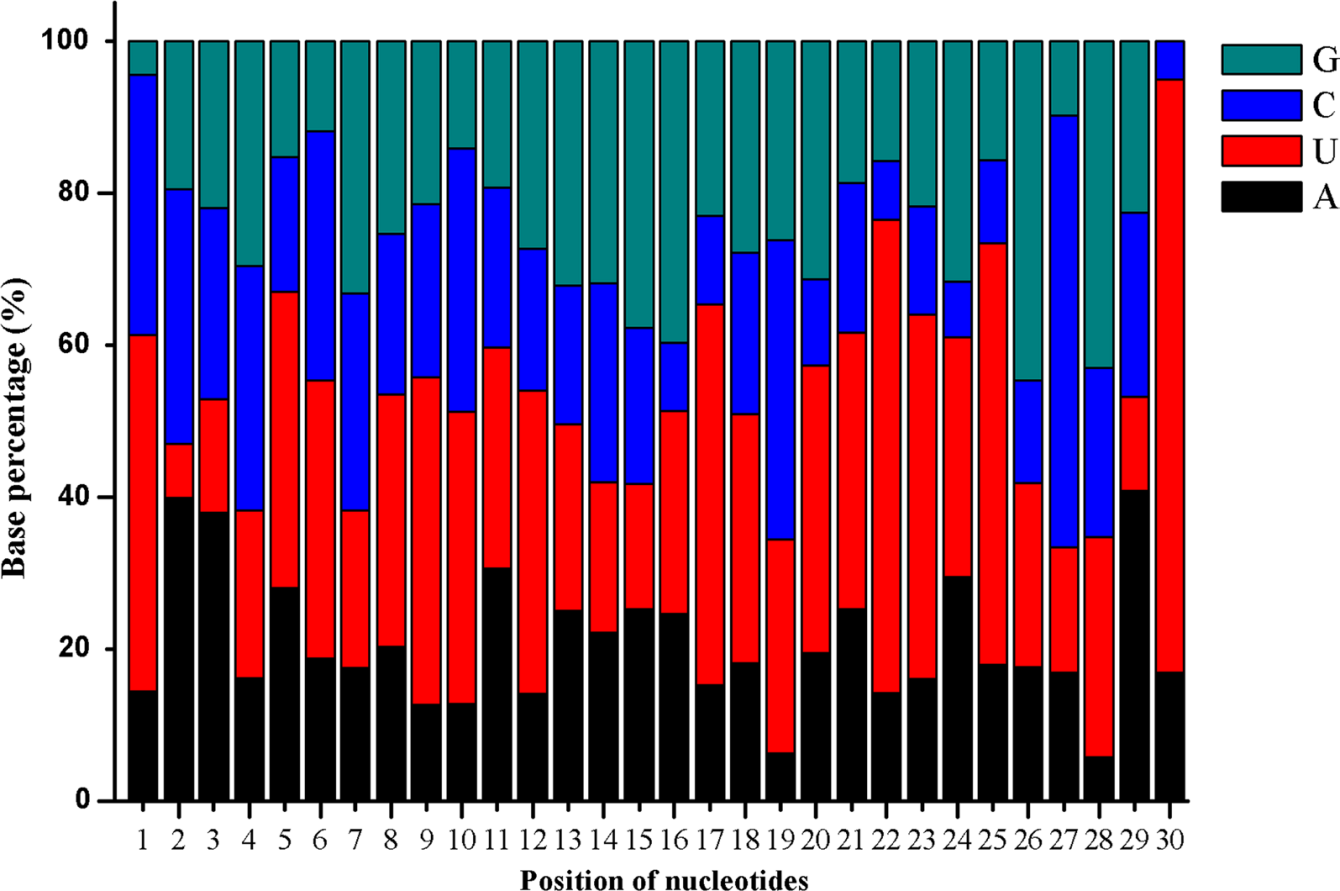
The position-specific nucleotide occurrence of *A. gossypii* mature miRNAs. Uracil dominated the first nucleotide position towards the 5′ end of miRNAs

Since the dominance of uracil at the first position of the 5′ terminus terminal is considered to be one of the conserved features of mature miRNAs [31], and given that the first base toward the 5′ end of miRNAs is known to play very important roles in the interaction between miRNAs and argonaute complexes [32], the position-specific nucleotide occurrence of the candidate miRNA sequences was analyzed. *A. gossypii* miRNAs showed a nucleotide bias towards uracil (U) at the first nucleotide position (Fig. 2). In addition, the base composition of the miRNAs at each position was analyzed, and the nucleotide U was the most abundant nucleotide at most of the positions; this was especially pronounced at positions 1, 17, 22, 23, 25, and 30 (Fig. 2).

### *A. gossypii* miRNAs differentially expressed following allelochemical treatments

**Fig. 3:**
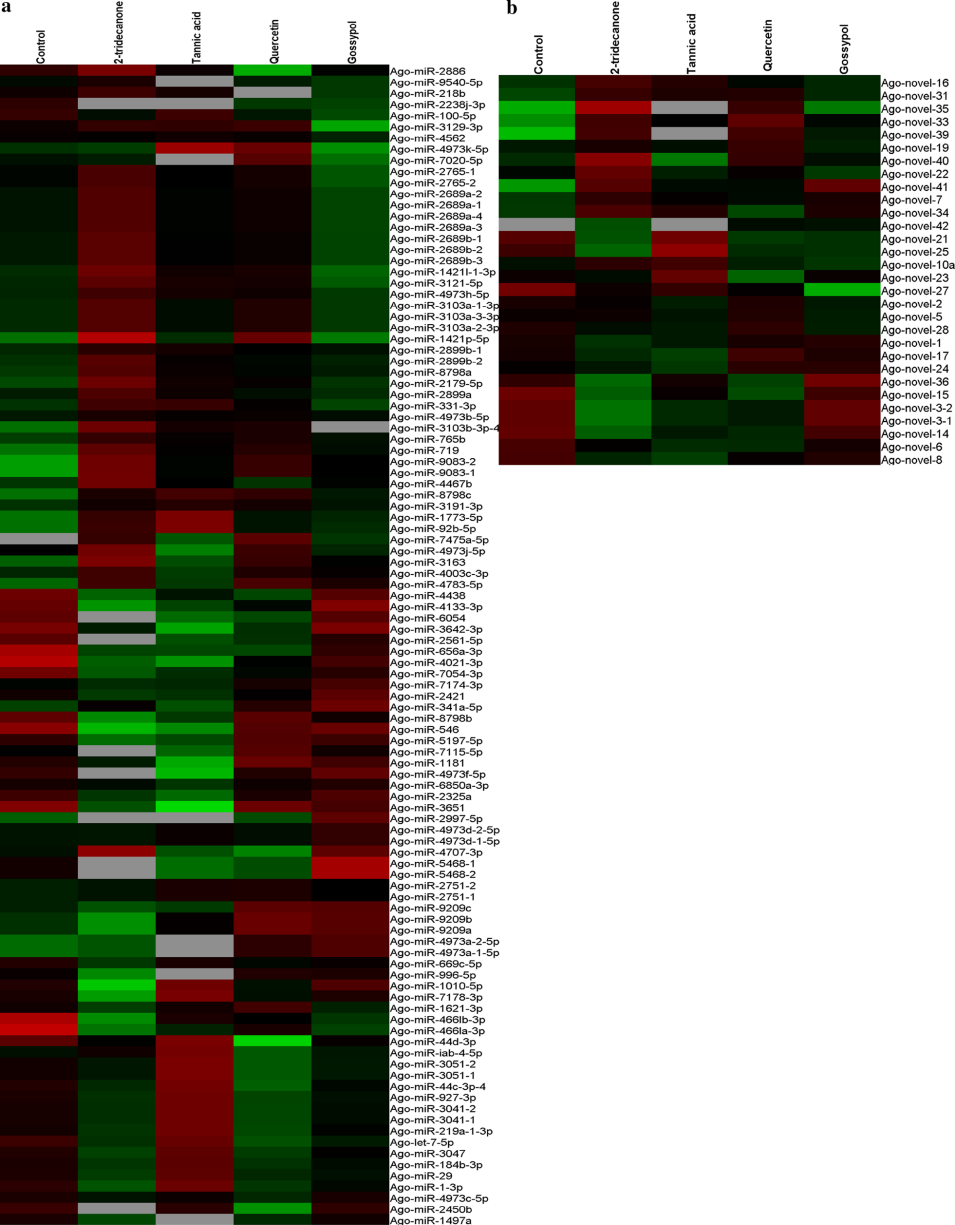
Differential expressions of *A. gossypii* miRNAs after feeding on various allelochemicals for 24 h. **a** Expression of conserved miRNAs. **b** Expression of novel miRNAs. *Green color* represents low expression levels and *red color* represents the high expression levels of the miRNAs

To identify the miRNAs that may play important roles in the responses of *A. gossypii* against plant alleochemicals, the differential expression of the identified miRNAs were analyzed using edgeR software. The expression values as assessed by miRDeep2 were used to analyze differential expression of *A. gossypii* miRNAs, and the miRNAs that had read counts of more than 10 in all five libraries were selected for differential expression analysis. Compared to the aphids fed on a 0.5 M sucrose solution (control), 134 miRNAs were found to be differentially expressed in the aphids fed on diet containing allelochemicals (Fig. 3). Of these, following for aphids feeding on gossypol, 2-tridecanone, quercetin, and tannic acid for 24 h, there were 33, 73, 59, and 42 differentially expressed miRNAs, respectively (Table 3). Interestingly, we found that most of the differentially expressed miRNAs were up-regulated in the aphids treated with gossypol, quercetin, and 2-tridecanone, but that the miRNAs from the tannic acid treated aphids possessed the tendency of down-regulation (Table 3).

**Table 3:** Number of miRNAs that were significantly differentially regulated after the different allelochemical treatments

Allelochemicals	Number of up-regulated miRNAs	Number of down-regulated miRNAs
2-tridecanone	42	31
Tannic acid	4	38
Quercetin	49	10
Gossypol	29	4

The expression levels of these miRNAs were dysregulated among five libraries. For instance, Ago-miR-2997-5p expression was low in the control and quercetin treatment libraries and was not detected in the 2-tridecanone or tannic acid treatment, but was up-regulated in the gossypol library (Fig. 3). Although the expression patterns of most of the miRNAs differed among the various allelochemical treatments, several miRNAs were consistently up/down-regulated in all four of the allelochemical treatment libraries. For instance, compared to the control, the expressions of some miRNAs (Ago-miR-8798a, Ago-miR-331-3p, Ago-miR-3191-3p, Ago-miR-1773-5p, Ago-miR-2179-5p, Ago-miR-92b-5p, Ago-miR-9083-2, and Ago-miR-719) were up-regulated in all four of the allelochemical treatments (Table 4). While the expression levels of Ago-let-7-5p, Ago-miR-100-5p, Ago-miR-44b-3p, Ago-miR-7054-3p, Ago-miR-4021-3p, Ago-miR-656a-3p, Ago-miR-4661a-3p, and Ago-miR-2238j-3p were down-regulated in all four treatment libraries in compare with the control (Table 5). It is worth nothing that Ago-miR-7475a-5p expression was not detected in the control library, but was expressed in each of the allelochemical treatment libraries (Table 4).

**Table 4:** The read counts and sequences of *A. gossypii* miRNAs that had increased expression following plant allelochemical treatment

miRNA name	miRNA sequence	Control	2-tridecanone	Tannic acid	Quercetin	Gossypol
Ago-miR-8798a	CGCGGTCGCCGCGCCGCC	116	199	117	154	162
Ago-miR-331-3p	GCCCCTGGTGGTCATGTTGGA	39	58	56	57	42
Ago-miR-3191-3p	GGGGGACGAGGTGGCCGAGCGGT	37	39	45	60	54
Ago-miR-1773-5p	GGGGGGAGGAGGAGGAGGA	19	41	75	36	39
Ago-miR-2179-5p	ATGCAAAATACATTTGTGTACT	27	66	32	45	35
Ago-miR-92b-5p	CGGGACGGCGAGGGTTGGGG	16	36	64	30	31
Ago-miR-719	CTCTCGGCCGTCGGCGCGGC	2	6	3	6	5
Ago-miR-9083-2	GGCCACGCCGCGCCGTCGG	1	5	2	5	4
Ago-miR-7475a-5p	CGCCACCGCCGCGCCGTCGT	0	6	2	13	5

**Table 5:** The read counts and sequences of *A. gossypii* miRNAs that had decreased expression following plant allelochemical treatment

miRNA name	miRNA sequence	Control	2-tridecanone	Tannic acid	Quercetin	Gossypol
Ago-let-7-5p	TGAGGTAGTTGGTTGTATAGT	2943	673	2378	811	1586
Ago-miR-100-5p	GACCCGTAGATCCGAACTTGTG	2501	803	1521	1171	962
Ago-miR-44b-3p	TGACTAGAGTTTATACTACCGA	1475	497	1375	606	835
Ago-miR-7054-3p	CCAACTTGGCAGCTTCTGA	158	17	25	52	95
Ago-miR-4021-3p	TAAGTATTTGGCTCTTGG	52	3	2	10	22
Ago-miR-656a-3p	CATATTATGGTCGTGAGTA	24	2	2	3	10
Ago-miR-466la-3p	TATAAATATTGTAGGTACC	23	1	2	5	3
Ago-miR-2238j-3p	TATGACGAGAGGGCAAAT	16	0	0	6	7

**Fig. 4:**
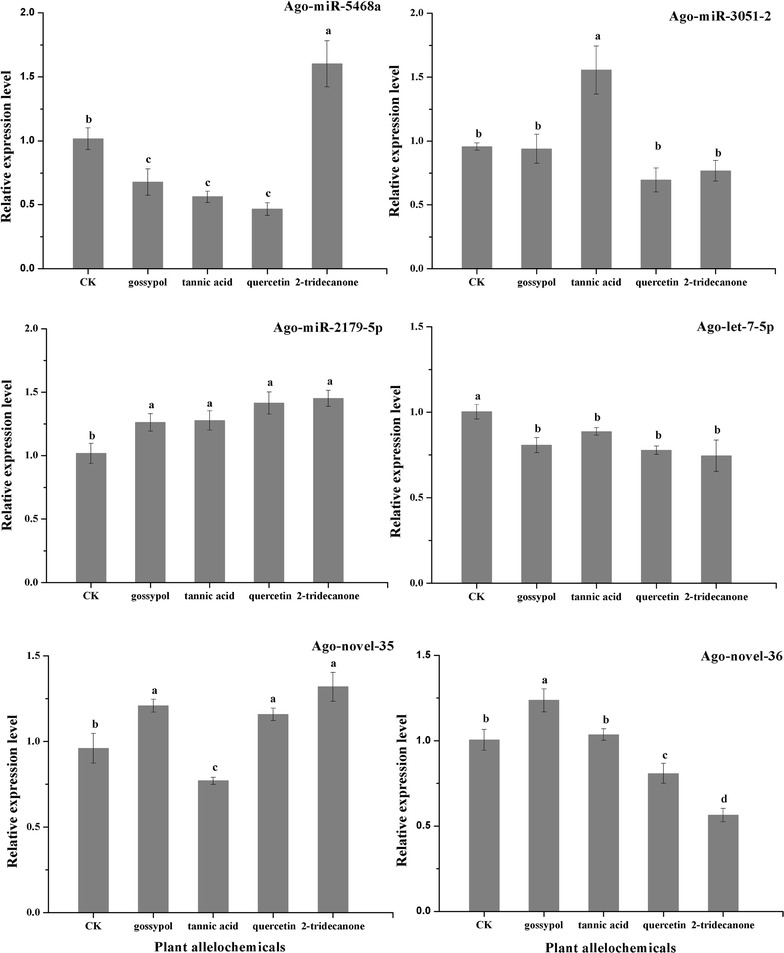
Differential expressions of miRNAs following plant allelochemical treatment. The results are presented as mean ± SD for three independent replicates. *Different letters* on the *bars* of the histogram indicate significant differences based on ANOVA followed by Tukey’s HSD multiple comparison test (*P* < 0.05)

To verify the expression analysis results from the sequencing experiments (read count), 6 differentially expressed miRNAs were selected, and their expression levels were measured via qRT-PCR analysis. Five of the selected miRNAs showed similar expression patterns as those assessed using sequencing read counts (Figs. 3, 4). In the sequencing results, the expression of Ago-miR-2179-5p was up-regulated, and Ago-let-7-5p was down-regulated in all of the allelochemicals treatment libraries, and the qRT-PCR analysis showed similar results (Fig. 4; Tables 4, 5). The qRT-PCR results for Ago-miR-3051-2 agreed with the sequencing results: it was highly expressed when apterous *A. gossypii* adults fed on an artificial diet containing 0.2% tannic acid (Figs. 3, 4). The expression level of Ago-miR-5468a as measured by qRT-PCR was different from that as assessed by the sequencing read count, the reason for this discrepancy is unknown.

### Prediction of the putative target genes of the miRNAs

To further understand the role of the various miRNAs identified in *A. gossypii*, the putative targets of these identified miRNAs were computationally predicted. The miRanda and RNAhybrid programs were used to identify the targets of these miRNAs from an *A. gossypii* transcriptome database. Combining the results of these two target prediction programs, a total of 5929 genes were putatively targeted by 236 miRNAs from *A. gossypii*. GO annotation of these target genes included the following categories: cellular components, molecular function, and biological process. About 49.8% of the predicted target genes were classified into the biological process category, including cellular process (19.4%), metabolic process (17.6%), and biological regulation (10.8%) (Additional file 2: Figure S2).

To characterize the potential roles of *A. gossypii* miRNAs in the defense responses against allelochemicals, we focused our attention to the predicted target genes that were likely to be involved in xenobiotic metabolism. Interestingly, we found that several miRNAs were targeted to genes that are known to play very important roles in insect responses to xenobiotic stress, including cytochrome P450s, acetylcholinesterases, glutathione S-transferases, sodium channel proteins, etc. (Table 6). Several miRNAs were predicted to have many target genes, and numerous genes were putatively targeted by multiple miRNAs. For instance, Ago-miR-4467a-1 and Ago-miR-4973-5p-11 were found have many predicted target genes, and CYP6J1 was putatively targeted by Ago-miR-656a-3p, Ago-miR-669c-5p, and Ago-miR-4172-3p (Table 6). In addition, some of these miRNAs, which were putatively targeted to xenobiotic metabolism-related genes, were differently expressed following allelochemical treatment, including Ago-miR-3191-3p, Ago-miR-8798a, and Ago-miR-656a-3p (Table 6; Fig. 3).

**Table 6:** Putative xenobiotic metabolism-related target genes of *A. gossypii* miRNAs

miRNA	Xenobiotic metabolism related target genes
Ago-miR-1-3p	G-protein coupled receptor
Ago-miR-341b-5p	Acetylcholinesterase, CYP6A8, CYP6A14
Ago-miR-656a-3p	CYP6J1, glutathione S-transferase sigma 1, carboxylesterase
Ago-miR-669c-5p	CYP6J1, glutathione S-transferase sigma 1
Ago-miR-1181	CYP6A13
Ago-miR-1332-3p	UDP-glucuronosyltransferase, CYP6A8, CYP6K1, CYP4C1, CYP6A2, CYP6A13
Ago-miR-1946a	Acetylcholinesterase, glutathione S-transferase omega 1
Ago-miR-2886	Acetylcholinesterase
Ago-miR-2899a	CYP18A1
Ago-miR-3163	Glutathione S-transferase sigma 1
Ago-miR-3191-3p	Carboxylesterase
Ago-miR-3575	CYP315A1, glutathione S-transferase sigma 2
Ago-miR-4172-3p	CYP6J1, glutathione S-transferase sigma 1
Ago-miR-4213-5p	Carboxylesterase, glutathione S-transferase sigma 2
Ago-miR-466la-3p	Carboxylesterase
Ago-miR-4467a-1	Sodium channel protein, calcium channel flower, CYP6A8, ryanodine receptor 44F, gamma-aminobutyric acid receptor, voltage-dependent calcium channel type A subunit alpha 1, nicotinic acetylcholine receptor alpha subunit
Ago-miR-4467a-2	CYP6A2, CYP6A8, esterase FE4, ryanodine receptor 44F
Ago-miR-4783-5p	Glutamate-gated chloride channel, voltage-dependent T-type calcium channel subunit alpha 1
Ago-miR-4973a-1-5p	Ryanodine receptor
Ago-miR-4973-5p-11	Ryanodine receptor 44F, sodium channel protein para, voltage-dependent L-type calcium channel subunit beta 2, Nic acetylcholine receptor alpha 2, Glutamate-gated chloride channel, Voltage-dependent T-type calcium channel subunit alpha 1
Ago-miR-4973 g-5p	Acetylcholinesterase, CYP6K1
Ago-miR-6236	Voltage-dependent calcium channel type A subunit alpha 1
Ago-miR-6850-3p-1	CYP6A8
Ago-miR-7475a-5p	Venom carboxylesterase-6
Ago-miR-8798a	CYP6A8
Ago-miR-8798c	Voltage-dependent calcium channel type A subunit alpha 1
Ago-novel-7	Sodium channel protein 60E, CYP6A8, UDP-glucuronosyltransferase
Ago-novel-10a	Ryanodine receptor 44F
Ago-novel-16	CYP18A1
Ago-novel-19	Acetylcholinesterase, CYP6A8, CYP6A2
Ago-novel-24	Acetylcholinesterase, CYP6A8
Ago-novel-28	Voltage-dependent calcium channel type A subunit alpha 1

### Validation of the target prediction

**Fig. 5:**
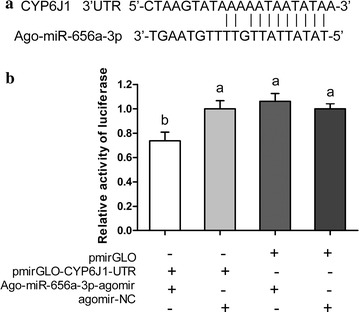
Interaction between Ago-miR-656a-3p and *CYP6J1* using a dual fluorescent reporter system. **a** Predicted target sites of Ago-miR-656a-3p in the 3′ UTR of *CYP6J1*. **b** Luciferase reporter assays performed by co-transfection of Ago-miR-656a-3p agomir with a luciferase reporter gene linked to the 3′ UTR of *CYP6J1*. Firefly luciferase activity was normalized to Renilla luciferase activity and then normalized to the activity of the control group. The mathematical operators of “+”and “−” mean add and subtract. *Different letters* on the *bars* of the histogram indicate significant differences based on ANOVA followed by Tukey’s HSD multiple comparison test (*P* < 0.05)

The target prediction results were validated by selecting a cascading of Ago-miR-656a-3p and *CYP6J1*. To determine whether or not Ago-miR-656a-3p could bind to the 3′ UTR of *CYP6J1* and suppress the expression of *CYP6J1*, the 3′ UTR of *CYP6J1* containing the target site of Ago-miR-656a-3p was inserted into a pmirGLO vector to yield a recombined vector, pmirGLO-CYP6J1-UTR. The results of dual luciferase reporter assay showed that the firefly luciferase activity normalized to Renilla was significantly reduced after pmirGLO-CYP6J1-UTR was co-transfected with Ago-miR-656a-3p agomir in comparison with the negative mimic control; while the co-transfection of Ago-miR-656a-3p agomir with control vector (pmirGLO) did not decrease the relative activity of luciferase (Fig. 5).

## Discussion

Allelochemicals are very important plant natural products that are known to play essential roles in plant defense responses to herbivores. Previous studies have shown that allelochemicals have great impacts on herbivorous insects. For example, detrimental effects were observed when *H. armigera* larvae were exposed to a high concentration of gossypol [33, 34]. Similarly, naringenin and quercetin have been reported to cause detrimental effects in the pea aphid, *A. pisum*, by effecting development, fecundity, and mortality [26]. As a very important polyphagous pest, cotton aphids encounter multiple allelochemicals in their life cycles, including gossypol, quercetin, and tannic acid. There is no doubt that allelochemicals have great effects on *A. gossypii*; for example, Gao et al. found that higher levels of gossypol adversely affected the development, longevity, and reproduction of *A. gossypii* [35]. miRNAs have been demonstrated to play very important regulatory roles in many biological processes over last few years. Therefore, it is reasonable to conjecture that small RNA molecules potentially function in regulatory roles in cotton aphid responses to plant allelochemicals. The identification and analysis of the expression profiles of miRNAs in allelochemical treated *A. gossypii* can potentially provide insight into the regulatory mechanisms underlying insect detoxification of plant allelochemicals. The present study was undertaken to identify the conserved and novel miRNAs of *A. gossypii* and to investigate the potential regulatory roles of these miRNAs in the metabolism of allelochemicals.

With the development of high throughput sequencing technology, small RNA sequencing has become a popular experimental approach to identifying miRNAs from a range of organisms. Sattar et al. [36] identified 102 miRNAs in *A. gossypii* fed on susceptible and resistant (*Vat* ^−^ and *Vat* ^+^) melon plants. In the present study, we sequenced five small RNA libraries and identified 293 miRNAs from *A. gossypii* fed on various allelochemicals; our sequencing data greatly expands the scope of the resources available to study *A. gossypii* miRNAs. The size distribution pattern of the clean reads revealed that the five libraries were dominated by 22 nt sequences (Fig. 1), a result consistent with the known characteristics of miRNA [37]. The overall length distribution observed of the *A. gossypii* miRNA in this study was similar to distributions observed in several other insect species, including *Blattella germanica* [38] and *Apis mellifera* [39].

Our expression analysis based on read counts showed that 134 miRNAs were differentially expressed in the allelochemical-treated aphids, as compared to the control aphids, which clearly suggests that allelochemicals affect miRNA expression, thus implying a possible role for miRNAs in the regulation of the metabolism of allelochemicals in *A. gossypii*.

Our results showed that several miRNAs were up/down regulated in all four allelochemicals libraries, and these miRNAs may play important roles in the metabolism of plant allelochemicals in *A. gossypii*. For instance, miR-92b was up-regulated in all four allelochemical libraries, and suggests that miR-92b may involve in the response of *A. gossypii* to plant allelochemicals. In other insect species, miR-92b has been reported to be involved in multiple biological processes. In *Drosophila*, miR-92b plays an important role in muscle development [40], and is essential for neuroblast self-renewal [41]. Meanwhile, miR-92b was classified as a stress responsive marker in *Eurosta solidaginis* [42].

In the present study, the expression levels of Ago-let-7-5p and Ago-miR-100-5p were down-regulated following allelochemical treatment. Let-7 and miR-100 are basic components of the let-7-complex (let-7-C), which is required for the development of normal morphology in *D. melanogaster* [43]. Given that allelochemicals can have great effects on insect development [26, 34, 35], the differential expression of let-7 and miR-100 might be attributable to the influence of plant allelochemicals. The similar phenomenon was also observed in the host adaption of *Myzus persicae* to nicotianae by the let-7 and miR-100 participating in regulating of the expression of *CYP6CY3* post-transcriptionally [44]. Further, Sattar et al. found that when apterous cotton aphids fed on *Vat* ^−^ melon with high susceptibility to aphids, the expression levels of let-7 and miR-100 were decreased in compare with the aphids that fed on resistant (*Vat* ^+^) melon [36]. These results suggest that let-7 and miR-100 might be involved in the metabolism of xenobiotics of *A. gossypii*.

To further understand the possible roles of miRNAs in the metabolism of plant allelochemicals, the putative target genes of the newly identified miRNAs were predicted, and many of the predicted target genes were annotated to be involved in multiple biological processes. In addition, we found that several miRNAs were predicted to target genes from families known to be important in the metabolism of xenobiotics [45, 46]. Combining these results of the target prediction with our differential expression analysis, we found that some of the miRNAs predicted to target these xenobiotic metabolism-related genes were among the differentially express miRNAs. This suggested that miRNAs may be involved in insect metabolism of plant allelochemicals by regulating the expression of xenobitic metabolism genes.

## Conclusions

A total of 292 miRNAs was identified from *A. gossypii*, and the expression analysis results demonstrated that the transcript levels of these miRNAs were changed depend on the plant allelochemicals feeding by *A. gossypii*. The results of target prediction suggest that miRNAs may be involved in the metabolism of plant allelochemicals of *A. gossypii*, and these results represent an important new small RNA genomics resource for further studies on this topic.

## Methods

### Cotton aphid strain and cell culture

The strain of *A. gossypii* used in this study was collected in 1999 from cotton fields in the Xinjiang Uygur Autonomous Region, China, and has been maintained in our laboratory for more than 15 years. The aphids were reared on cotton seedings in controlled conditions of 20–23 °C, 60% relative humidity, and a photoperiod of 16:8 h (L:D), as described previously [47]. The mammalian HEK293T cell line was maintained at 37 °C under 5% CO_2_ in DMEM high-glucose medium (Gibco) containing 10% fetal bovine serum (Gibco).

### Chemicals

Gossypol, 2-tridecanone, quercetin, and tannic acid were purchased from Sigma-Aldrich (St. Louis, MO, USA). Sucrose was purchased from Beijing Solarbio Science & Technology Co., Ltd (Beijing, China).

### *In vitro* feeding assay

Sterilized glass tubes open at both ends (3 cm in length, 2 cm diameter) were used for the in vitro feeding assays. One end of each tube was covered by two layers of parafilm, with the following solution sandwiched between the two parafilm layers (artificial diet): 200 µl of a 0.5 M sterile sucrose solution that contained 0.2% gossypol, 2-tridecanone, quercetin, and tannic acid). One hundred healthy aperous adults were gently placed into the tube with a brush, and the tube was sealed with a piece of Chinese art paper with solid glue. Aphids were allowed to feed on this artificial diet for 24 h. The same system and solution, minus the alleochemicals, was used as a control. Following the 24 h of feeding, the live aphids were collected for RNA extraction.

### RNA extraction and sequencing

Five samples of apterous *A. gossypii* adults collected from the feeding assays were used for the preparation of the small RNA libraries. Total RNA was isolated with TRIzol® reagent (Invitrogen, Carlsbad, CA, USA) following the manufacturer’s instructions. The purity and the concentration of the RNA were assessed with a NAS-99 spectrophotometer (ACTGene, USA), and RNA integrity was evaluated with analysis on a 1% (w/v) agarose gel. About 10 µg of total RNA was isolated on a 15% denaturing polyacrylamide gel, and small RNA molecules ranging from 18 to 30 nt in length were purified and then ligated with 3′ and 5′ adapters. The ligated products were then reverse transcribed into cDNA with SuperScript II reverse transcriptase (Invitrogen, Carlsbad, CA, USA) following the manufacturer’s protocol, and the resulting cDNA was amplified with PCR (15 cycles). Amplified cDNA products were purified with agarose gels and sent to the Beijing Genome Institute Inc. (China) for high-throughput sequencing with the Illumina Hiseq 2000 platform.

### Identification of miRNAs from sequencing data

*A. gossypii* miRNA candidates were identified using miRDeep2 software [48]. Raw sequencing reads from the five libraries were submitted as input into miRDeep2, and the data from each library were analyzed separately. The miRDeep2 analysis was performed using the default options and settings. After trimming the adaptor sequences and discarding rRNA, tRNA, snRNA, and snoRNA, as well as the sequences containing polyA tails from the raw reads, the small RNAs between 18 and 30 nt in length were selected for further analysis. The *Acyrthosiphon pisum* genome sequence was used as the reference genome, since the *A. gossypii* genome sequence was not available at the time of analysis.

### Expression profile of miRNAs in five libraries

In order to find the miRNAs that may play very important roles in the response of cotton aphids against plant alleochemicals, the differential expression of the identified miRNAs was analyzed. The read counts of the newly identified miRNA were analyzed using edgeR software; edgeR version 3.10.2 is available in Bioconductor version 3.1 (http://www.bioconductor.org/packages/release/bioc/html/edgeR.html) [49]. edgeR is a Bioconductor software package for analyzing the differential expression of digital gene expression data. Briefly, an overdispersed Poisson model is used to account for variability, and empirical Bayes methods are used to moderate the degree of overdispersion across the transcripts. The Benjamini-Hochberg method was used to adjust for multiple testing [50]. Only those miRNAs with a fold change ≥2 and a false discovery rate (FDR) <0.05 were considered to be significant.

### Target gene prediction and analysis

Since the complete genome sequence of *A. gossypii* was not available at the time of analysis, the *A. gossypii* transcriptome database (unpublished) was used to predict the targets of the sequenced *A. gossypii* miRNAs. Two miRNA target prediction software programs were used, each with the default settings: miRanda (http://www.microrna.org/) [51] and RNAhybrid (http://bibiserv2.cebitec.uni-bielefeld.de/rnahybrid/) [52]. The miRNA target genes commonly predicted by both programs were selected for further analysis. The predicted target genes were then aligned using the BLASTX program from NCBI (http://blast.ncbi.nlm.nih.gov/Blast.cgi) (e value cut-off used was 1.0E−5), and these genes were mapped and annotated by BLAST2GO [53]. The GO terms from this analysis were used to define the functional categories of the predicted miRNA target genes.

### Quantitative real-time PCR

To validate the miRNA data obtained through our deep sequencing experiment, 4 conserved and 2 novel miRNA candidates were selected, and their expression was confirmed with quantitative real-time PCR (qRT-PCR) analysis. Aperous adults fed on an artificial diet (with 0.2% allechemicals) for 24 h were used for total RNA isolation; total RNA was extracted using miRNeasy Mini Kit (Qiagen, Germany) following the manufacturer’s protocol. First strand cDNA was synthesized from 2 μg of total RNA using miScript II RT kit (Qiagen) following the manufacturer’s instructions. SYBR Green Master Mix (miScript SYBR Green PCR Kit, Qiagen) was used for miRNA expression assays, and qRT-PCR was performed as previously described [15]. Three biological replicates, with three technical replications for each, were evaluated for each sample. Analysis of the qRT-PCR data was carried out using the 2^−∆∆Ct^ method of relative quantification [54]. As an endogenous control, U6 snRNA was used to quantify the expression level of miRNA. The primers used for the qRT-PCR analysis are listed in Additional file 2: Table S1.

### Vector construction and dual luciferase reporter assay

The target prediction results were validated by selecting Ago-miR-656a-3p and *CYP6J1* that has a target site of Ago-miR-656a-3p in the 3′ UTR. The *CYP6J1* 3′ UTR sequence was synthesized by GenePharm Co. Ltd (Shanghai, China), and was inserted into the pmirGLO vector, generating the pmirGLO-CYP6J1-UTR target construct. The agomir (mimics) of Ago-miR-656a-3p was synthesized by GenePharm Co. Ltd (Shanghai, China). The HEK293T cells were cultured in a 24-well plate and were transfected with target plasmids and agomir of Ago-miR-656a-3p or agomir-NC using a Calcium Phosphate Cell Transfection Kit (Beyotime, Nanjing, China) according to the manufacturer’s instruction. Each well contained 0.5 μg of the plasmid, with the final concentration of miRNA agomir reaching 100 nM/L. Luciferase assays were performed by using the Dual-Glo® Luciferase Assay System (Promega) at 24 h post-transfection. Normalized firefly luciferase activity (firefly luciferase activity/Renilla luciferase activity) was compared to that of the control pmirGLO Vector. The mean of the relative luciferase expression ratio (firefly luciferase/renilla luciferase) of the control was set to 1. For each transfection, luciferase activity was averaged from five replicates.

## Additional files

**Additional file 1.** Length distribution of unique small RNAs from A. gossypii identified by deep sequencing.

**Additional file 2: Table S1.** Primers used in the qRT-PCR analysis. **Figure S1.** The common and unique distribution of identified *A. gossypii* miRNAs among the five libraries. S01: CK; S02: 2-tridecanone; S03: Tannic acid; S04: Quercetin; S05: Gossypol. **Figure S2.** The GO annotation of *A. gossypii* miRNAs target genes.

## Abbreviations

miRNAs: microRNAs
UTRs: untranslated regions
P450: cytochrome p450
PCR: polymerase chain reaction
qRT-PCR: quantitative real-time PCR
U: uracil
let-7-C: let-7-complex
