# Supplementary material for: Identification of microRNAs and their response to the stress of plant allelochemicals in Aphis gossypii (Hemiptera: Aphididae)
Source: BMC Mol Biol. 2017 Feb 16;18:5. doi: 10.1186/s12867-017-0080-5 (PMC5311835; doi:10.1186/s12867-017-0080-5)
Supplement: Supplementary file 2 — Additional file 2: Table S1. Primers used in the qRT-PCR analysis. Figure S1. The common and unique distribution of identified A. gossypii miRNAs among the five libraries. S01: CK; S02: 2-tridecanone; S03: Tannic acid; S04: Quercetin; S05: Gossypol. Figure S2. The GO annotation of A. gossypii miRNAs target genes. [file 12867_2017_80_MOESM2_ESM.docx]

**Table S1** Primers used in the qRT-PCR analysis

|  | |
| --- | --- |
| miRNA | Forward Primer (5’-3’) |
| Ago-novel-36 | CCACGCGTCCGGTTGTCG |
| Ago-miR-3051-2 | GCGCACAAGGAACGTTAA |
| Ago-miR-5468-1 | GCAGCCTTGGAGTGTGGT |
| Ago-novel-35 | GCACGTCCACTCCCCAGCT |
| Ago-miR-2179-5p | CGGAGCATGCAAAATACAT |
| Ago-let-7-5p | GCGGTGAGGTAGTTGGTTGT |
| U6 | GCTTCGGCAGHACATATACTAA |


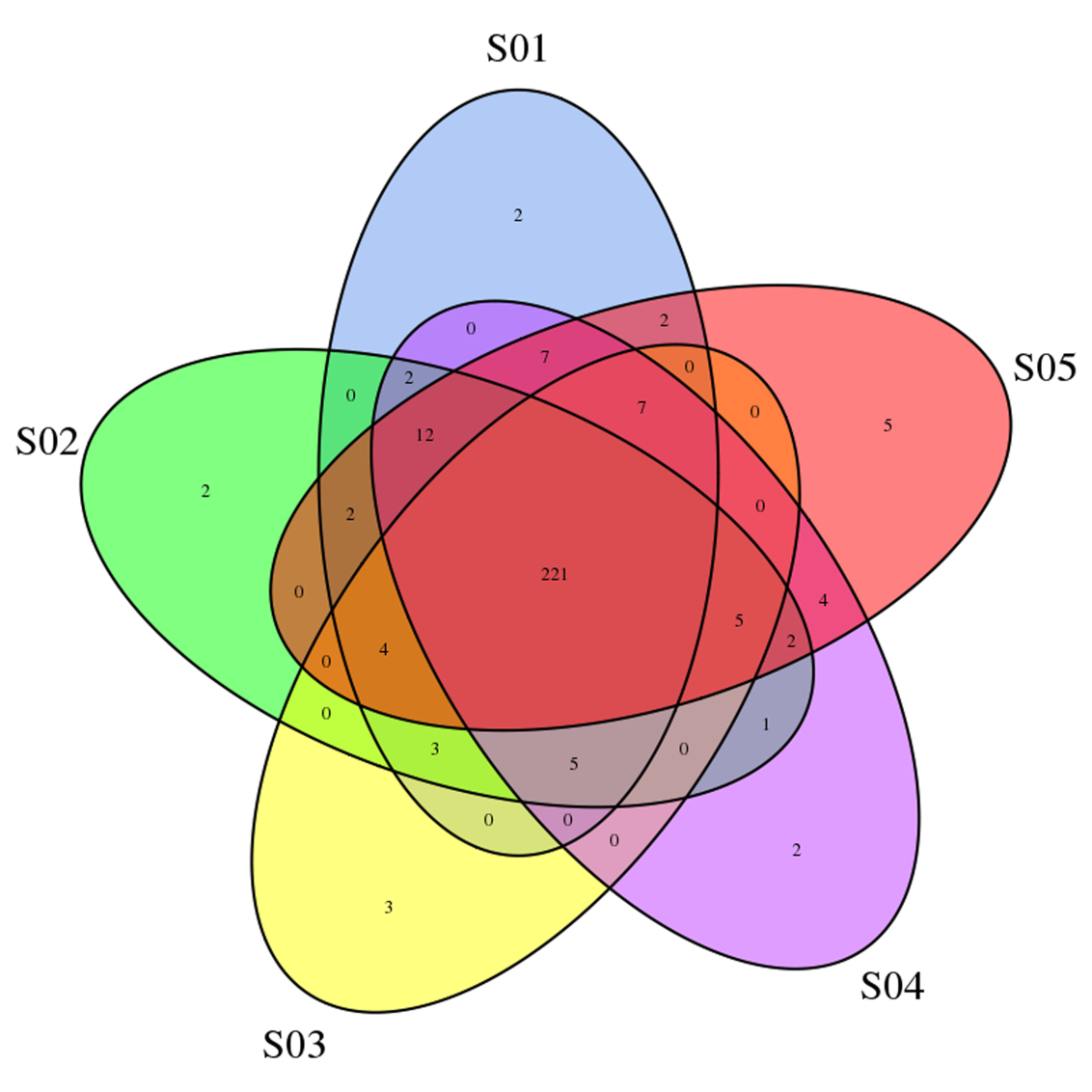


**Figure S1** The common and unique distribution of identified *A. gossypii* miRNAs among the five libraries. S01: CK; S02: 2-tridecanone; S03: Tannic acid; S04: Quercetin; S05: Gossypol.


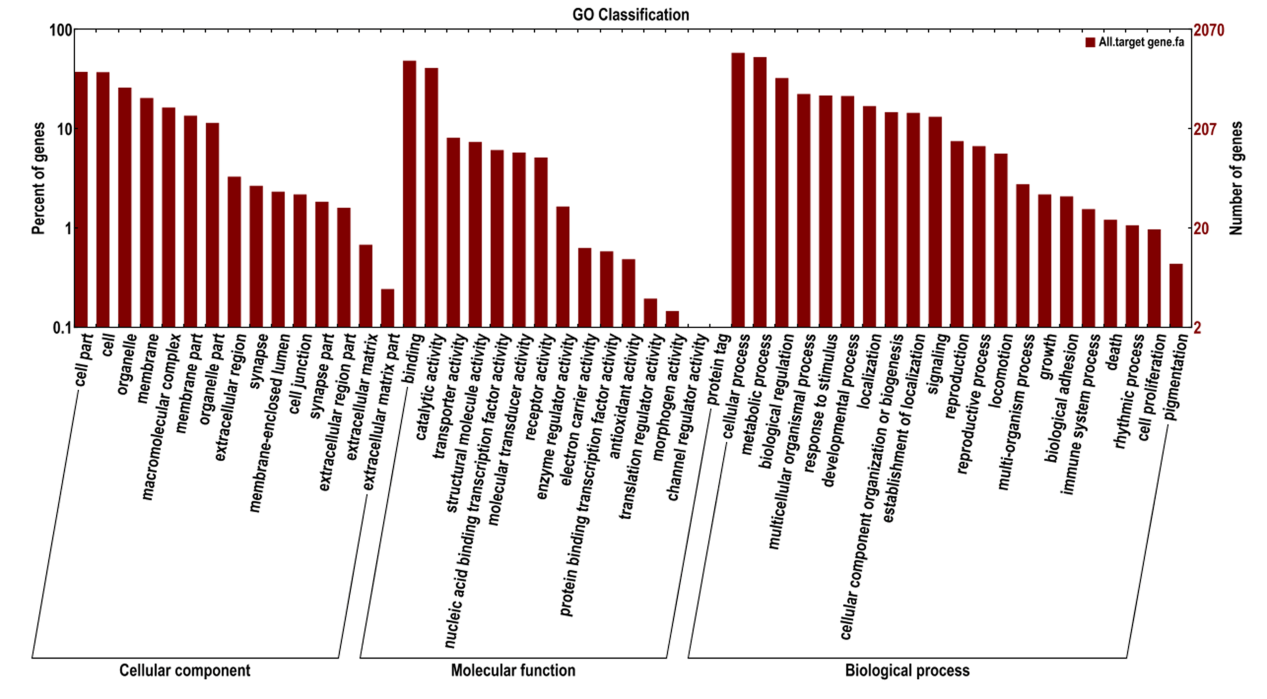


**Figure S2** The GO annotation of *A. gossypii* miRNAs target genes.
